# Young and vulnerable: Spatial-temporal trends and risk factors for infant mortality in rural South Africa (Agincourt), 1992-2007

**DOI:** 10.1186/1471-2458-10-645

**Published:** 2010-10-26

**Authors:** Benn KD Sartorius, Kathleen Kahn, Penelope Vounatsou, Mark A Collinson, Stephen M Tollman

**Affiliations:** 1 MRC/Wits Rural Public Health and Health Transitions Research Unit (Agincourt), School of Public Health, Faculty of Health Sciences, University of the Witwatersrand, Johannesburg, South Africa; 2 Centre for Global Health Research, Epidemiology and Global Health, University of Umeå, Umeå, Sweden; 3 INDEPTH Network, Accra, Ghana; 4 Swiss Tropical and Public Health Institute, Basel, Switzerland

## Abstract

**Background:**

Infant mortality is an important indicator of population health in a country. It is associated with several health determinants, such as maternal health, access to high-quality health care, socioeconomic conditions, and public health policy and practices.

**Methods:**

A spatial-temporal analysis was performed to assess changes in infant mortality patterns between 1992-2007 and to identify factors associated with infant mortality risk in the Agincourt sub-district, rural northeast South Africa. Period, sex, refugee status, maternal and fertility-related factors, household mortality experience, distance to nearest primary health care facility, and socio-economic status were examined as possible risk factors. All-cause and cause-specific mortality maps were developed to identify high risk areas within the study site. The analysis was carried out by fitting Bayesian hierarchical geostatistical negative binomial autoregressive models using Markov chain Monte Carlo simulation. Simulation-based Bayesian kriging was used to produce maps of all-cause and cause-specific mortality risk.

**Results:**

Infant mortality increased significantly over the study period, largely due to the impact of the HIV epidemic. There was a high burden of neonatal mortality (especially perinatal) with several hot spots observed in close proximity to health facilities. Significant risk factors for all-cause infant mortality were mother's death in first year (most commonly due to HIV), death of previous sibling and increasing number of household deaths. Being born to a Mozambican mother posed a significant risk for infectious and parasitic deaths, particularly acute diarrhoea and malnutrition.

**Conclusions:**

This study demonstrates the use of Bayesian geostatistical models in assessing risk factors and producing smooth maps of infant mortality risk in a health and socio-demographic surveillance system. Results showed marked geographical differences in mortality risk across a relatively small area. Prevention of vertical transmission of HIV and survival of mothers during the infants' first year in high prevalence villages needs to be urgently addressed, including expanded antenatal testing, prevention of mother-to-child transmission, and improved access to antiretroviral therapy. There is also need to assess and improve the capacity of district hospitals for emergency obstetric and newborn care. Persisting risk factors, including inadequate provision of clean water and sanitation, are yet to be fully addressed.

## Background

Infant mortality is an important health indicator of a population given its strong link to socio-economic status (SES), health service access and quality, and maternal health.

In the absence of vital events registration, health and socio-demographic surveillance (HDSS) data provide a valuable source for estimating mortality rates, trends and risk factors. HDSS sites implementing the verbal autopsy (VA) to determine probable cause of death are often the only means in most developing and many middle-income countries to observe cause-specific mortality of a population on a longitudinal basis and are a valuable tool for assessing trends in burden of disease [1,2].

Diarrhoea, pneumonia, malnutrition and malaria are the leading causes of death among infants in low income countries [3,4]. Birth asphyxia and neonatal sepsis are responsible for most neonatal deaths [3]. These diseases, that can be largely prevented or effectively treated at relatively low cost, cause almost 95% of preventable infant and child deaths [1]. HIV/AIDS has emerged as a major cause of death among infants in recent years, though in few countries outside of Africa [5].

In 1990, there was a 20-fold difference in the rate of infant deaths between sub-Saharan African and industrialized countries (180 versus 9 deaths per 1000 live births). In 2000, this difference had increased to 29-fold with mortality rates of 175 and 6 per 1000 children respectively [6]. This is because many sub-Saharan African countries have seen reversals in child mortality trends in recent years due to HIV/AIDS. In 2007, approximately 420 000 children became infected with HIV [7], mostly through mother-to-child transmission (MTCT) [8,9] in resource poor settings particularly sub-Saharan Africa. Kahn et al showed a doubling of child mortality due to HIV in a rural South African population (Agincourt sub-district) between 1992 and 2003 from 39/1000 person-years to 77/1000 [10]. Garrib et al in 2006 found very high levels of infant mortality in another rural area of South Africa, 67.5 per 1000 person-years, with HIV/AIDS estimated as the single largest cause of death in the under-5 age-group (41% of deaths) [11]. Thus interventions to reduce infant and child mortality are urgently required. A study in Zambia estimated that the cost per averted infection was approximately US$890 [12]. According to a study in Barbados the lifetime cost of treating an HIV infected child is US$ 8,665 [13]. This is much lower than estimates from the US where the cost for perinatally infected infants was USD 113,476 for 9 years of survival, US$ 151,849 for 15 years, and US$ 228,155 for 25 years [14]. According to a study in the Ivory Coast, the mean cost of treatment was € (euros) 254 per child-year for infected children, €108 more than the mean cost of treatment for HIV-negative children born to HIV-positive mothers (a 74% increase in treatment costs) [15]. Thus despite the costs associated with HIV/AIDS prevention among young children [16,17], lifetime treatments costs of HIV infected infants are far higher; hence preventive measures need to be prioritized and targeted to those at high risk in poor, resource limited settings.

Effective interventions such as prevention of mother to child transmission (PMTCT) are available. A comprehensive approach to PMTCT can reduce transmission rates to below 2% [18-20]. Yet health care access and inequity remain widespread problems in economically disadvantaged areas [21]. Mpumalanga Province in northeast South Africa was an important destination for refugees fleeing the civil war in Mozambique from 1983 onwards. A formal peace agreement was signed in 1992, yet despite voluntary repatriation programmes, by 2000 it was estimated that more than 200,000 former Mozambican refugees were still inhabitants in the province [22]. A study by Hargreaves et al [23] demonstrated higher mortality rates among children from former Mozambican refugee households when compared to those from South African-headed households in the Agincourt sub-district. They concluded that lack of legal status and poorer SES of Mozambican refugees partly explains this disparity.

Inequalities in health outcomes or access to services and benefits can occur across space and time. In some situations this can reflect a compositional effect with variations merely reflecting the different groups that inhabit different locations [24]. However, certain inequalities in child health outcomes are avoidable and unjust. These may reflect underlying inequities in the distribution of wealth, resources and social privilege in a given society, rather than an individual's choice or behaviour. To be fair, society must strive to achieve equal opportunities for all children regardless of parental status (education, SES) and geographical location. High-quality services for children that bridge the social divide are an important means of achieving equity goals. If South Africa is to achieve the Millennium Development Goals by 2015, including MDG 4 to reduce child mortality, then there is need to scale-up coverage rapidly with access to high quality health care and social support, particularly in the most poor and marginalised communities [25]. When population-wide intervention programmes are too costly to implement, it becomes necessary to target such efforts to high risk areas where adverse health events are the most likely to occur [26]. To address inequity in child survival, service planners need to understand the underlying socio-demographic profile and other factors contributing to high risk. Spatial-temporal mapping of high risk communities identifies those with greatest need, rather than those that are easiest to reach [27]. This provides evidence on where to target interventions for greatest impact [28] and generates hypotheses on the determinants of increased risk.

With the development of Markov Chain Monte Carlo (MCMC) methods and Bayesian software such as WinBUGS, geostatistical spatial-temporal modelling has increasingly been applied in epidemiological research [29], especially with regards to malaria risk and transmission. Gemperli et al (2004) carried out a Bayesian spatial analysis of infant mortality in Bali that confirmed well-known risk factors and found a spatial pattern of infant mortality that showed a clear relationship with established foci of malaria transmission [30-32]. Despite growing applications of spatial methodology in malaria research, fewer studies have analysed spatial variations in population dynamics including all-cause and cause-specific mortality, with little or no work on longitudinal data collected in relatively small geographic areas covered by health and socio-demographic surveillance.

Many individual and household level factors have been identified as key determinants of infant and child mortality. Since objects in close proximity are often more alike, common exposures (measured or unmeasured) may influence mortality similarly in households of the same geographical area, introducing spatial correlation in mortality outcomes. Repeated measurements on individuals and households are also expected to be correlated in time. Standard statistical methods assume independence of outcome measures, for example mortality data. Ignoring this correlation introduces bias in the risk analysis as the standard error of the risk factors is underestimated, thereby overestimating significance. Bayesian geostatistical models relax the assumption of independence by, for example, incorporating random effects to measure spatial correlation as a function of distance between locations.

The aim of this study was to assess changes in infant mortality patterns in rural northeast South Africa over time, determine mortality risk factors and produce cause-specific mortality maps to identify high risk areas. These insights can provide guidance on the best allocation of limited resources to reduce infant mortality in this and similar areas of the country.

## Methods

### Study area and population

**Figure 1:**
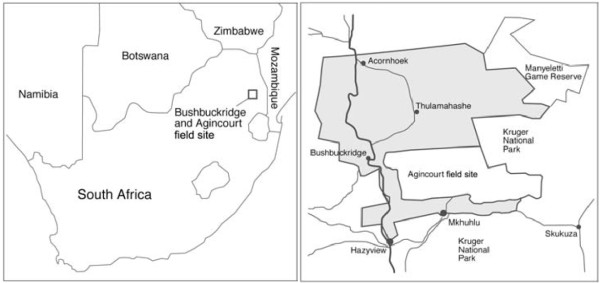
**Location of the Agincourt HDSS site **[33]**, South Africa**.

The Agincourt health and socio-demographic surveillance system (HDSS), established in 1992, extends over an area of about 400 km^2 ^and consists of 21 villages with approximately 11,700 households and a population of 70,000 people at the end of 2007 (Figure 1). A full geographic information system (GIS) covers all households within the site and is updated annually. For these analyses the study population consisted of all infants who were either born or migrated into the site between 1992 and 2007 and who either survived or died in their first year of life.

### Outcome measures

A verbal autopsy (VA) was conducted on every death to determine its probable cause [34]. Interviews administered by trained lay fieldworkers were assessed independently by two physicians to determine probably cause-of-death. Where consensus could not be reached, a third independent medical assessment was made. The VA was first validated in the mid-1990s [35] and again in 2006 with particular reference to HIV/AIDS related mortality. International Classification of Diseases (ICD-10) was used to classify main or underlying, immediate and contributory causes of death. For this study, cause-specific analysis was limited to main causes from 1992-2006 as VA's had not yet been assessed for 2007.

### Explanatory variables

Covariates included: infant demographic variables (gender, nationality); 5-year time periods; maternal factors (former refugee status, age at pregnancy, death in first year of child's life, education); fertility factors (parity, birth intervals, sibling death); household mortality experience, socio-economic status (SES) and food security; distance to health facility; antenatal clinic attendance; and household elevation (climatic proxy). Every two years since 2001, an asset survey was conducted in all households within the HDSS [36]. Information on living conditions and assets, building materials of main dwelling, water and energy supply, ownership of modern appliances and livestock, and means of transport available were recoded (one being higher SES and zero lower status), summed to give an overall score for a household, and then used to construct wealth quintiles for SES ranked by increasing score from most to least poor.

### Statistical analysis

The negative binomial is an alternative for the commonly used Poisson distribution, often regarded as the default distribution for integer count data. The Poisson assumes that expected mean equals its variance. The negative binomial differs from the Poisson distribution in that it allows for the variance to exceed the mean. Since the negative binomial distribution has one more parameter than the Poisson distribution, the second parameter is used to adjust the variance independently of the mean. Our data displayed evidence of being highly overdispersed and thus the negative binomial model was chosen.

A preliminary negative binomial regression analysis was carried out to assess the relationship between infant mortality and each covariate. Covariates significant at the 10% level (without substantial missing values) were then incorporated into the multivariate model.

The multivariate Bayesian negative binomial model was fitted in WinBUGS to examine the association between the significant covariates and all-cause infant mortality. Observation dates were used to calculate the person-days contributed by each infant (offset). Spatial random effects were used at a village level to take into account spatial correlation. Temporal random effects were also used at yearly intervals to account for temporal correlation. Village specific random effects were modelled via a multivariate Gaussian process (multivariate Gaussian distribution with covariance matrix expressed as a parametric function of distance between pairs of village centroid points) [37]. Standard Bayesian autoregressive (AR) approaches, with priors for the AR(1) and AR(2) processes defined by Schotman [38] and Zeller [39] respectively, as well as a Poisson generalized autoregressive moving average (GARMA) approach [40], were tested to model the temporal random effects. Various order models for the AR and MA terms were assessed and the one that best fitted the data was used. MCMC simulation was employed to estimate the model parameters [41]. Further details of the statistical modelling approach are given in the appendix.

### Model assessment and validation

Deviance Information Criterion (DIC) [42] was used as the first step in comparison of model fit and the one giving the lowest DIC was chosen. Models were then also validated by fitting the models for 1992-2006 and predicting outcomes for all infants in 2007. Credibility intervals were constructed and the model providing the best predictions (along with low DIC) were used as the final model. The negative binomial models, particularly the AR(1) and AR(2) to model the temporal random effect, provided the lowest DIC (8618.07 and 8617.34 respectively) by some margin when compared to other approaches such as GARMA. In Bayesian statistics, a credible interval is a posterior probability interval which is used for interval estimation, in contrast to point estimation (confidence intervals). In other words, the credibility interval refers to the distribution of parameter values while a confidence interval pertains to estimates of a single value. In this study the negative binomial AR(2) predicted the outcome much better than the AR(1) model based on these Bayesian credibility intervals. Thus the AR(2) process was used in the final multivariate model.

### Risk maps

A baseline model was used that included no covariates but a constant and site-specific (village centroid) random effect. All identifying features (village centroids, geographic boundaries) were removed, and the prediction area expanded irregularly (~740 km^2^) to double the normal size, in order to ensure confidentiality and avoid stigmatizing of villages. The HIV/TB map is not shown for this reason. Simulation-based Bayesian kriging [43] at prediction points (regular grid) within the site was used to produce maps of mortality risk for the whole HDSS site. Model estimates were exponentiated to represent incidence rate ratios (IRR).

### Software

Data extraction and management was done using Microsoft SQL Server 2005. The analysis was carried out in STATA 10.0, WinBUGS and R. The predictions of the fitted spatial models were mapped in MapInfo Professional 9.5.

## Results

### Demographic profile of study sample

Between 1992 and 2007 31,804 infants were either born or migrated into the Agincourt HDSS. Of these, 26,000 (81.8%) were born within the site and half (50.4%) were female. Just under two-thirds were South African citizens (20,375; 64.2%) and a little over one-third were born to Mozambicans (11,356; 35.8%). There were 737 infant deaths (2.3%) giving an overall mortality rate of 24.7 per 1,000 person years; of these, 175 deaths were within the perinatal period and 202 within the neonatal period.

### Cause of death (1992-2006)

The top causes of death among infants, as assessed by verbal autopsy, were HIV/TB (n = 116), acute diarrhoea or malnutrition (n = 91), acute respiratory infection (ARI) or pneumonia (n = 82) and septicemia (n = 20). In total 300 infant deaths were attributed to infectious and/or parasitic causes. During 1992-2006, 230 infants (33.6%) had an unknown cause of death.

### Temporal trends by cause

**Figure 2:**
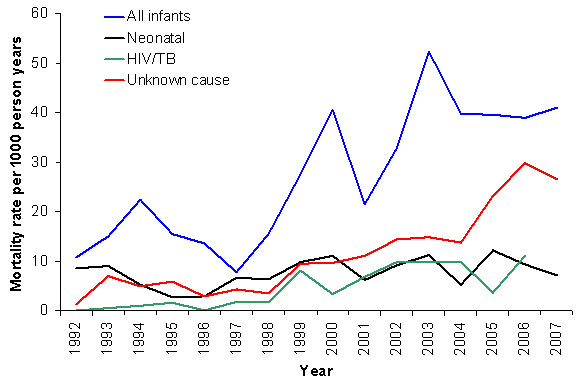
**All-cause neonatal and infant mortality rates per 1,000 person years, Agincourt sub-district 1992-2007**.

**Figure 3:**
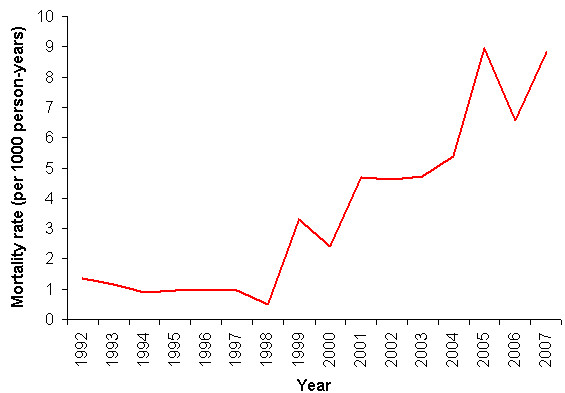
**Mortality rate of mothers dying in infants' first year per 1000 infant person-years, Agincourt sub-district, 1992-2007**.

There was a significant increasing trend in the infant mortality rate over the study period (IRR = 1.09, 95%CI: 1.05-1.12, p < 0.001) (Figure 2). A significant increasing trend (at 10% level) was also observed for all-cause neonatal (first 28 days of life) mortality rate (IRR = 1.04, 95%CI: 1.00-1.08, p = 0.068), particularly from 1996 onwards. Mean time to death among neonates was 4.49 days (SD 6.05) indicating that most occur in the perinatal period (first 7 days of life). Between 1992 and 2006 the infant mortality rate due to HIV/TB significantly increased from 0 to 10.95 deaths per 1000 person years (IRR = 1.27, 95%CI: 1.17-1.38, p < 0.001), the increase commencing from about 1998. No significant changes were observed for infant deaths due to acute diarrhoea or malnutrition and ARI or pneumonia. A significant increasing trend was observed with infant deaths attributed to unknown (R99) causes (IRR = 1.27, 95%CI: 1.19-1.36, p < 0.001), particularly from 1998 onwards. A significant (IRR = 1.14, p < 0.001) and striking increase in the mortality rate of mothers dying in the infants' first year was also observed (Figure 3), again from 1998 onwards.

### Univariate analysis

Later time period, higher number of cumulative household deaths, death of previous sibling and mother dying in the first year of infant's life were large and highly significant risk factors for infant mortality (Table 1). Male gender and increasing birth parity were also found to be significant risk factors. Breast feeding had a protective influence on all-cause as well as diarrhoea and malnutrition-related infant mortality (Table 1). Increasing infant weight at birth also had a significantly protective effect. High (post-secondary) level of maternal education, mother attending antenatal clinic and increasing number of antenatal clinic visits were found to be significantly protective. Mozambican origin of mother was not found to be a risk factor for all-cause infant mortality. However, mother having arrived from Mozambique post 1992 was found to be a significant risk factor for death due to infectious and/or parasitic causes. Increasing distance to nearest health facility was not a significant risk factor and no differential health care access was observed by nationality (South African versus Mozambican). Mother being a migrant was found to be significantly protective and, conversely, increasing number of months spent resident in the site per year by the mother was found to be a risk. Further, migrant mothers were found to be significantly more educated than mothers permanently resident in the study site (p < 0.001) and came from households with a significantly higher SES (p = 0.0025). No significant difference was found in antenatal clinic attendance between permanent and migrant mothers.

**Table 1:** All-cause univariate risk factor analysis for infant mortality in the Agincourt sub-district, 1992-2007

Factor	n	IRR	p-value	signif
*Temporal*				
1 year continuous	31,804	1.23	<0.001	*
				
5 year period	31,804			
1992-1996	10,744	1.00		
1997-2001	10,624	4.61	<0.001	*
2002-2006	10,436	10.76	<0.001	*
				
*Demographic*				
Male gender	31,804	1.86	0.006	*
				
Mother refugee status	29,068			
South African citizen	18,746	1.00		
Mozambican origin	10,322	1.12	0.638	
				
*Breast feeding and birth weight*				
Breast fed	23,890/25,697	0.21	<0.001	*
Breast fed (diarrhoea & malnutrition)	23,890/25,697	0.38	0.001	*
				
Increasing birth weight (kilograms)	15,235^β^	0.42	<0.001	*
				
*Maternal*				
Mother Mozambican in-migrated post 1992 ^α^	10,322	4.89	0.004	*
				
Mother status	31,041			
Mother in same household	27,076	1.00		
Mother not in household	2,488	0.01	<0.001	*
Mother death	1,477	5.79	<0.001	*
				
Mother residency status				
Permanent (> = 6 months in site)	28,852	1.00		
Migrant	25,200	0.45	0.059	#
Other	2,035	0.36	0.067	#
				
Increasing number of months resident during the previous 12 months	28,962	1.11	0.001	*
				
Mother died in child's first year	91/31,804	65.72	<0.001	*
				
Mother age at pregnancy	27,981	0.99	0.595	
				
Mother education	16,971			
None or primary	6511	1.00		
Secondary	9561	0.90	0.677	
Tertiary	899	0.13	<0.001	*
				
*Paternal*				
Father died in child's first year	59/31,804	0.69	0.834	
Father died before birth	57/31,804	0.98	0.993	
				
*Household head*	26,034			
Male gender	16,625	0.86	0.331	
Mozambican origin	9,972	1.04	0.808	
Age (years)	25,660			
18-29	2,995	1.00		
30-49	13,143	0.93	0.785	
50-64	6,486	1.39	0.243	
65+	3,036	1.43	0.272	
				
*Household morbidity and mortality*				
Cumulative number of household deaths in year of birth (continuous)	31,804	14.64	<0.001	*
None	26,444	1.00		
1	4,048	35.51	<0.001	*
2-3	1,232	109.34	<0.001	*
4+	80	131.47	<0.001	*
				
Number of household admissions in year of birth (continuous)	2,895	1.55	0.093	#
None	2,392	1.00		
1-2	402	1.51	0.464	
3+	101	17.80	0.041	*
				
*Fertility*				
Birth parity (continuous)	25,083	1.22	0.170	
1	25,083	1.00		
2-3	16,526	2.32	0.001	*
4+	7,663	0.55	0.313	
Preceding birth interval	8,421	1.05	0.001	*
Post birth interval	7,199	0.51	<0.001	*
Previous birth stillborn	318/27,981	16.71	<0.001	*
Previous sibling died	945/27,981	7.48	0.001	*
Preceding interval sibling death	413	1.23	0.413	
Mother attended antenatal clinic	24,928	0.06	<0.001	*
Number of antenatal clinic visits	17,305	0.84	<0.001	*
				
*Socio-economic status of household*				
SES absolute score (quintiles)	9,397			
Most poor	1,584	1.00		
Very poor	1,801	0.81	0.540	
Poor	1,971	0.91	0.780	
Less poor	2,019	0.91	0.780	
Least poor	2,022	0.98	0.940	
				
*Food security status of household*				
Predicted food shortage in coming year	3,481			
Same amount of food	1,122	1.00		
More food	377	0.31	0.091	#
Less food	1,982	1.24	0.597	
				
*Distance to nearest health facility*				
Minimum distance to health facility (straight-line) from household	25,749			
< 5 km	24,023	1.00		
> = 5 km	1,726	1.18	0.607	
				
Minimum distance to health facility (network) from village centroid	31,804			
< 5 km	1,220	1.00		
> = 5 km	30,584	1.28	0.684	
				
*Climatic*				
Elevation (meters) - rainfall proxy	30,583			
350-399	2,554	1.00		
400-449	14,202	0.43	0.072	#
450-499	4,120	0.25	0.009	*
500-549	7,202	0.17	<0.001	*
550-599	2,505	0.46	0.226	

Total sample size (n = 31,804)

α: infectious and parasitic deaths only

β: birth weight data only available for this number of infants

* significant at the 5% level; # significant at the 10% level

**Figure 4:**
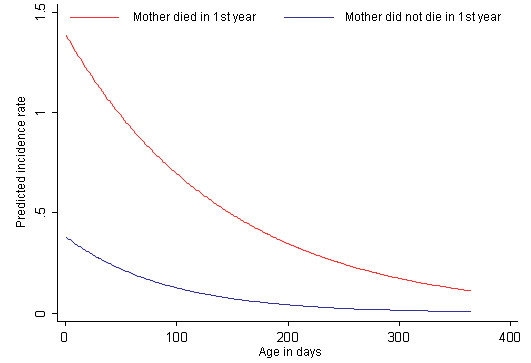
**Predicted infant mortality incidence rate by day of life and mother status in first year, Agincourt sub-district**.

There was a strong non-linear reduction in the probability of death as the infant progresses to the end of their first year (Figure 4). This risk over time was much higher for those infants whose mother died in their first year, and remained elevated for the remainder of the first year.

A significant increase in the number of years of maternal education as well as antenatal clinic attendance was observed over the study period (both p < 0.001). However, significant increases in the number of mothers dying in their infants' first year, number of maternal deaths (< = 42 days after infants date of birth), as well as other household deaths over time was also observed (all p < 0.001).

Almost a third (30.2%, n = 91) of mothers who died in the infants' first year died of HIV/TB; this was a significant risk factor for infant mortality (IRR = 164.7, p < 0.001). Approximately 44% of the mothers that died in the infant's first year died of unknown causes, many probably unclassified HIV-related deaths.

Household water supply consisting of raw natural water (river, pond or dam) was a risk factor (IRR = 16.50, p = 0.010) for deaths due to acute diarrhoea and malnutrition, although numbers were small. Mother being of Mozambican origin also proved to be a significant risk factor for infant death due to diarrhoea or malnutrition (IRR = 1.66, p = 0.019).

### Multivariate analysis

Later year of birth, mother dying in the infant's first year, higher number of cumulative household deaths and previous birth being stillborn remained highly significant in the all-cause multivariate model (Table 2). Following multivariate adjustment, large IRR values were again observed for mother death in the infant's first year, and cumulative household deaths. Death of previous child was also a significant risk factor at the 10% level. There was more temporal than spatial correlation in the final spatial-temporal model (0.44 versus 0.09). The spatial-temporal model estimated the range (distance at which spatial correlation ceases) to be 5,224 metres (95%: 1,805-21,420) meters. AR(1) and AR(2) parameters were between -1 and 1 indicating stationarity.

**Table 2:** All-cause multivariate risk factor analysis for infant mortality in Agincourt sub-district, 1992-2007, using Bayesian geostatistical and temporal models

	Spatial model		Temporal model		Spatial-temporal model	
Covariate	IRR [95%CI]	**signif **^**α**^	IRR [95%CI]	**signif **^**α**^	IRR [95%CI]	Signif ^α^
Later year of birth	1.09 [1.06,1.13]	*	1.20 [1.03,1.42]	*	1.25 [1.07,1.53]	*
Cumulative household deaths	12.45 [9.41,16.31]	*	12.52 [9.47,16.26]	*	12.59 [9.53,16.49]	*
Male gender	1.09 [0.84,1.4]		1.09 [0.83,1.4]		1.09 [0.84,1.4]	
Mother died in infant's first year	49.82 [7.85,204.7]	*	49.62 [8,189.9]	*	51.11 [8.49,200.8]	*
Pregnancy parity	0.91 [0.80,1.05]		0.94 [0.82,1.08]		0.94 [0.82,1.06]	
Previous child died	2.33 [0.99,4.86]	#	2.07 [0.93,4.07]	#	2.02 [0.89,3.96]	#
Previous birth stillborn	8.13 [2.08,22.99]	*	6.24 [1.55,16.99]	*	6.29 [1.56,17.85]	*
						
Constant (b0)	-3.24 [-4.60,-1.82]		-0.10 [-2.09,1.65]		-0.63 [-3.08,0.97]	
σ^2 ^(spatial)	9.15 [4.29,18.33]		---		0.09 [0.03,0.22]	
σ^2 ^(temporal)	---		0.42 [0.14,1.05]		0.44 [0.14,1.11]	
						
DIC ^β^	8680.07		8617.10		8617.34	

α: * significant at the 5% level; # significant at the 10% level

β: Deviance Information Criterion

### Risk maps

#### All-cause

**Figure 5:**
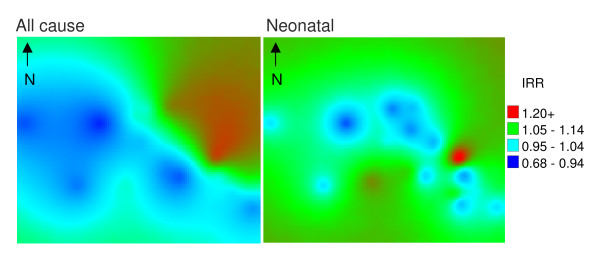
**Maps of all-cause and neonatal mortality risk within the Agincourt sub-district 1992-2007, based on baseline models without covariates**.

**Figure 6:**
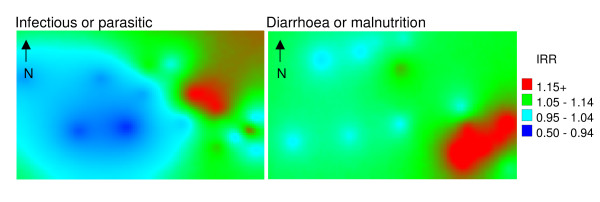
**Maps of selected cause-specific infant mortality risk within the Agincourt sub-district, 1992-2006, based on baseline models without covariates**.

Figure 5 shows all-cause and neonatal mortality risk. Note that with increasing distance from locations with observed mortality (i.e. villages), the standard error of the prediction increases. The map of all-cause infant mortality risk reveals the highest risk to be among villages bordering the Kruger National Park to the east of the site, running from the upper central towards the south-east. Distinct foci of high mortality risk can be identified in two villages in particular. Neonatal mortality displayed a similar pattern with 2 distinct foci of higher risk. One of these foci is in close proximity to a health facility. Figure 6 shows a distinct pattern of higher infectious and/or parasitic causes, including HIV/TB, towards the east of the site, again with distinct foci of higher mortality in former refugee settlements. Acute diarrhoea and malnutrition showed a distinct cluster of higher mortality in the south-east. The pattern of ARI or pneumonia infant mortality risk was less distinct, though two foci could be observed to the east of the study area.

## Discussion

The results indicate a worsening of infant mortality: year of birth was significantly associated with infant mortality and risk of death increased over the study period. The increase was particularly from 1998 onwards, and can be largely attributed to the HIV epidemic and its impact on mortality in the study area [9,44], both direct (vertical transmission of HIV) and indirect (death of a caregiver). Mother's death in infant's first year was a major risk factor in this study, as was higher numbers of cumulative household deaths. Results confirmed the importance of other known risk factors [23,30]. The protective association between increasing maternal education and infant mortality has been previously described [30,45] and is possibly a result of better health awareness and utilization of health facilities [46], longer birth intervals [47], and higher income which improves infants' health through ability to purchase goods and services [48]. A significant association of higher household SES was however not observed in this study. This has been shown elsewhere and may be explained by that fact that unlike endogenous maternal and demographic factors that substantially influence an infant's risk of death, the effects of SES factors on mortality increase as the child gets older due to exogenous factors which parents have more control over [49].

We examined health service access with respect to primary health care generally and antenatal care specifically. Distance to nearest primary health care facility was not a risk factor in this study. Antenatal clinic attendance and number of ANC visits was significantly protective, with no difference between South Africans and former Mozambican refugees. These finding suggest that factors other than geographic access may be key to understanding the risks associated with health care utilisation. These could include quality of care, level of available care (primary versus secondary), cost and social barriers. In South Africa, primary health care for children under the age of six is free, as is antenatal care. However, financial costs associated with transport and opportunity costs associated with lengthy waiting time [50] are some of the barriers described in this setting [51,52]. Twine el al showed that the poorest households were less likely to apply for social support grants than those in higher socioeconomic strata due to barriers such as distance from government offices, lack of official documentation and education of caregiver and household head [51].

A recent study in Kenya found that, despite significant spatial variations in child mortality, these were not correlated with distance to health facilities [53]. They concluded that geographic access to curative services did not influence population-level mortality given the density of health facilities in Kenya. They also suggest that when distance access targets are met, further improvements in child survival can only be achieved through renewed investigation of the social, behavioural and quality-of-care factors that may obstruct access to health care services. Similarly in rural South Africa, there is urgent need to evaluate and assure a high level of health service quality, assess and strengthen referral patterns for emergency obstetric, infant and child health care, and identify other barriers to accessing these and other government services.

Mothers' physical presence or absence had a significant impact on infant mortality: mother a temporary migrant (largely work-related) proved significantly protective, while conversely increasing number of months per year spent resident by the mother in the rural site was a risk. Brockerhoff [54] describes how maternal rural-urban migration may affect children through three types of living arrangement: children may remain in the village as foster-children in the care of their fathers or other relatives; children may accompany or follow their mothers to towns or cities; and children born after migrant mothers settle in an urban area may remain there through the first few years of life. (Note that in this study, infants born to mothers in urban areas would not be captured onto the HDSS database unless they later migrated into the rural household). Bledsoe et al, [55] reviewing evidence from West Africa, suggest that while fostered children may be disadvantaged compared to biological children (in terms of access to health care and nutrition), they may still be better off than if they had accompanied their migrant mothers. By staying home, these children avoid exposure to infectious diseases during a vulnerable period of their life, have continued access to economic resources of a non-migrant father, and benefit from remittances received from the migrant mother [55] - as well as better health care, nutrition and enhanced maternal health knowledge [56]. In our study, migrant mothers had significantly higher education and came from households with significantly higher SES which may explain the protective effect of mothers' migration. According to Collinson et al, [57] since 1997 there has been an increasing trend in the number of temporary female labour migrants in the Agincourt sub-district, a poor area with limited employment opportunities with resulting pressures to migrate and remit wages back to the rural household.

The spatial distribution showed marked geographical differences in all-cause mortality risk, indicating variation even within a relatively small area. The highest infant mortality risk was in those villages on the eastern border of the site. Much of this spatial distribution can be explained by the migration patterns of former Mozambican refugees (who constitute about a third of the Agincourt HDSS population) who entered South Africa via the Kruger National Park, a wild game conservation area situated between the eastern border of the site and Southern Mozambique. They remained a vulnerable group, poorer in more isolated villages with less infrastructure and generally further away from health facilities, with poor access to water and sanitation as well as labour markets and legal rights [58]. However, our study indicates that the all-cause infant mortality risk pattern is not being driven by former refugee status alone, a finding supported by Hargreaves et al [23] who found no difference in mortality rates between South African and former Mozambican refugee infants between 1992 and 2000, despite significant differences in the 1-4 year age group. Multiple factors are driving the observed all-cause spatial risk pattern, including Mozambican origin of mother for certain infectious causes, maternal death in first year of infant's life, lower maternal education, poor quality of and limited access to neonatal care, poor antenatal clinic attendance, and increased vulnerability of households with a high mortality burden. These factors should be better elucidated and quantified in order to contribute meaningfully to policy and programmes.

With regard to the geographical distribution of infectious infant deaths (particularly HIV/TB) there was a distinct spatial pattern of mortality with an increasing gradient towards the east of the site where communities appear to have increased risk and suitable interventions need to be directed accordingly. One village in particular had a significantly higher mortality rate (all-cause and HIV) when compared to all other villages. Diarrhoea and malnutrition-related mortality was clustered in the south east of the site suggesting greater problems with clean water and sanitation, services that need to be assessed and addressed by local government. Breastfeeding had a protective effect on all-cause as well as diarrhoea and malnutrition-related infant mortality (Table 1). Breastfeeding protects infants through decreased exposure to contaminated water and food, optimal nutrition, and improved resistance to infection however there is risk of HIV transmission through breast milk. In South Africa, Ministry of Health policy on breastfeeding by HIV positive mothers has evolved in response to emerging research [59]; current recommendations are to breastfeed exclusively during the first 6 months with administration of anti-retrovirals to HIV positive mothers [60], especially those with low CD4 counts. Mothers or infants receiving highly active anti-retroviral therapy (HAART) prophylaxis should continue prophylaxis for one week after breastfeeding has ended [60]. Infant mortality due to diarrhoea, malnutrition and their interaction is a complex problem in poor, HIV prevalent African settings. Addressing this requires a multifaceted approach including provision of clean water and sanitation, promoting infant nutrition, and strengthened primary care services for mothers and infants to reduce the risk of HIV transmission through breast milk [61].

Addressing health inequities in populations is a major challenge [62], and research that documents and quantifies inequities is needed to inform policies to close health gaps in the developing world. Evidence on reducing inequities within countries is growing; successful approaches include those that improve geographic access to health interventions in poor communities, subsidize health care and health inputs for the poor, and empower poorer communities [63]. The results of our study indicate the need for interventions in villages to the east of the site, many of which have a large proportion of former refugees, to reduce the higher burden of infant deaths due to infectious and parasitic causes. HAART for HIV began in 2007 in this district and its impact cannot thus be captured during the time frame of this study. This research does, however, provide useful insight into spatial-temporal mortality patterns before HAART rollout and will allow post-rollout assessment of its impact on infant mortality. Such evaluation has the potential to identify areas needing improved access to treatment, specifically prevention of mother-to-child transmission and anti-retroviral therapy.

Of concern is the high number of neonatal deaths (particularly in the perinatal period), their gradual increase over the study period, and the highest risk area being in close proximity to a health facility. This suggests problems of service quality rather than geographic access, and highlights the need to assess and improve the capacity of sub-district health facilities for antenatal, emergency obstetric and newborn care; improve coverage of deliveries by skilled birth attendants; and advise mothers on appropriate care-seeking for sick babies. Part of the perinatal mortality burden observed may relate to maternal HIV since the same village experienced highest risk for neonatal and infant mortality. A meta-analysis by Brocklehurst et al [64] in 1998 found an association between maternal HIV infection and adverse perinatal outcomes, including low birth weight and pre-term delivery.

A limitation of the study is the potential to miss infant deaths, particularly neonatal deaths, which would underestimate the overall infant mortality burden. Infants that are born and then die during the 12 months between HDSS census update rounds may not be reported, particularly if the mother migrated out of the household; similarly, death among in-migrant infants who die before they are enumerated in the annual household census may be missed. However, infant death ascertainment has improved in the study site [36], and the proportion of infants who were in-migrants decreased significantly over time, reducing the bias towards the end of the study period. Determination of cause of death through verbal autopsy is more problematic for diseases that have less specific symptoms such as HIV/AIDS [65]. The prevalence of HIV infection in a population and the resulting rate of HIV-associated co-morbidity and death due to malnutrition in children, for example, may affect the performance (such as specificity) of the tool. Thus it is likely that the HIV burden is underestimated due to the misclassification of deaths as AIDS-related conditions such as malnutrition or diarrhoea, or there being placed in the "unknown cause" category. The significant increase in number of infant deaths attributed to unknown causes since the late 1990s (Figure 2) is concurrent with the rise in HIV-related mortality in the area. Levels of stigma associated with HIV are high in South Africa, particularly prior to the introduction of HAART. The ability to make a diagnosis on VA depends, in large part, on the quality of information provided by the respondent. This may have been compromised in some cases in an effort to disguise HIV as a likely cause of death, partly explaining the increase in unknown causes.

## Conclusion

By estimating the true spatial distribution of the infant mortality burden in rural northeast South Africa, this study has shown variation across a relatively small geographical area. The approach used Bayesian geostatistical models in order to assess risk factors, correctly estimate the standard errors (significance) of these risk factors and produce smooth maps of infant mortality risk from spatially correlated longitudinal mortality data in a health and socio-demographic surveillance system. Findings indicate the need for interventions targeted at villages with excess infant mortality risk due to both a direct and indirect impact of HIV. Essential interventions include improved prevention of mother-to-child transmission programmes, and antiretroviral therapy for HIV positive mothers to ensure their survival during their infants' critical first year(s) of life. From our study, it is clearly inadequate to consider maternal health separately from infant and neonatal health. This is consistent with other studies which showed that maternal health directly affects infants' health [66]. Policy should thus have greater emphasis on interventions targeting the mother-infant pair. We also conclude that the non-random clustering of infant mortality due to diarrhoea and malnutrition in the south-east part of the site represents a breakdown in basic services (or, indeed, their absence); there is hence need to assess and improve water and sanitation in these villages. The high levels of perinatal mortality, in some instances in close proximity to health facilities, is of concern, indicating need to strengthen the capacity of sub-district facilities for emergency obstetric and newborn care. Recommendations from this study will have applications to other similar rural settings within South Africa and potentially beyond.

## Appendix: Statistical Model

Let Y_it _and p_it _be the status and probability of mortality of an infant i in year of birth t. We assume that Y_it _arises from a negative binomial distribution, that is Y_it _~NegBin[p_it_, r], where p_it _*is *the probability that child *i *at location *si *is dead and *r *is the parameter that quantifies the amount of extra Poisson variation. We modelled the probability of death [p_it_] as follows:

1. logit (p_it_) = β_0 _+ βX_*it *_+ φ_*it *_(multivariate spatial model)

2. logit (p_it_) = β_0 _+ βX_*it *_+ α_*t *_(multivariate temporal model)

3. logit (p_it_) = β_0 _+ βX_*it *_+ φ_*it *_+ α_*t *_(multivariate spatial-temporal model)

4. logit (p_it_) = β_0 _+ φ_*it *_(spatial kriging model) i.e. constant and spatial random effect with no covariates

where β_0 _is the incidence rate where all covariates are zero (i.e. the constant), X_it _denotes the covariates, β is the vector of regression coefficients, φ_*it *_the village-specific random effect, μ_*i *_the individual level random effect and α_*t *_the temporal random effect. Following a Bayesian model specification, noninformative normal prior distributions were adopted for the regression coefficients β and an informative (based on estimates from Stata) and non-informative gamma prior distribution for the over-dispersion parameter *r *were adopted and tested [lower DIC dictating which was used]. We assume that φ_*it *_has a multivariate normal distribution, φ_*it *_~ MVN (0,Σ), with variance-covariance matrix Σ. We also assume an isotropic stationary spatial process, where Σkl = σ_w_^2 ^exp(-φ*d*_*kl*_), *d*_*kl *_is the Euclidean distance between villages *k *and *l*, σ_w_^2 ^is the geographical variability known as the sill, φ is a smoothing parameter that controls the rate of correlation decay with increasing distance and measures the range of geographical dependency. A noninformative gamma prior was adopted for phi [φ], which is the smoothing parameter that controls the rate of correlation decay, as well as uniform prior with a distribution between φ min and φ max [67]. Both approaches were tested and the approach providing the best fit was then used. The range is defined as the minimum distance at which spatial correlation between locations is below 5%. This distance can be calculated as 3/*u *meters. The second order year level autoregressive temporal random effect (α_t_), for t = 1 to 16 years, was modelled as a normal distribution with mean α_mean _[t = 3,..,16] = ρ0 + ρ[1]*α[t-1] + ρ[2]*α[t-2] and a noninformative gamma distribution for the variance parameter. The first two autoregressive terms were specified as α_mean _[1] <- ρ0 + l[1] and α_mean _[2] <- ρ0 + ρ[1]*α[1] + l[2]. Noninformative normal prior distributions were adopted for the ρ and l coefficients [34].

MCMC simulation was applied to fit the models. We ran a single chain sampler with a burn-in of 5000 iterations. Convergence was assessed by running the simulation until the Monte Carlo error for each parameter of interest was less than 5% of the sample standard deviation. The chains thereafter were sampled every single iteration until a sample size of 10,000 had been attained.

## Competing interests

The contents of this paper and the data used for it have not been published elsewhere. The paper is also not in press or under review elsewhere, nor has a similar paper been written by anyone else using the same data and methods.

## Authors' contributions

All authors have read and approved the final manuscript. BKD Sartorius: conception and design, data extraction, data analysis, drafted the manuscript. KK: acquisition of funding, conception and design, policy implications, reviewed manuscript. PV: acquisition of funding, conception and design, statistical support, reviewed manuscript. MAC: conception and design, reviewed manuscript. SMT: acquisition of funding, conception and design, reviewed manuscript

## Pre-publication history

The pre-publication history for this paper can be accessed here:

http://www.biomedcentral.com/1471-2458/10/645/prepub
